# Urinary markers of the alternative and lectin complement pathway are increased in IgA vasculitis nephritis

**DOI:** 10.1093/ckj/sfad236

**Published:** 2023-09-14

**Authors:** Julien Marro, Andrew J Chetwynd, Jennifer Hawkes, Sarah J Northey, Louise Oni

**Affiliations:** Department of Women's and Children's Health, Institute of Life Course and Medical Sciences, University of Liverpool, Liverpool, UK; Department of Women's and Children's Health, Institute of Life Course and Medical Sciences, University of Liverpool, Liverpool, UK; Centre for Proteome Research, Institute of Systems, Molecular and Integrative Biology, University of Liverpool, Liverpool, UK; Department of Women's and Children's Health, Institute of Life Course and Medical Sciences, University of Liverpool, Liverpool, UK; Department of Women's and Children's Health, Institute of Life Course and Medical Sciences, University of Liverpool, Liverpool, UK; Department of Women's and Children's Health, Institute of Life Course and Medical Sciences, University of Liverpool, Liverpool, UK; Department of Paediatric Nephrology, Alder Hey Children's NHS Foundation Trust Hospital, Liverpool, UK

**Keywords:** complement, Henoch–Schonlein purpura, HSP, kidney, paediatrics

## Abstract

**Background:**

IgA vasculitis (IgAV) is the most common form of childhood vasculitis. Nephritis (IgAVN) occurs in 50% of patients and 1–2% progress to chronic kidney disease stage 5. The pathophysiology of nephritis remains largely unknown, but recent evidence suggests that the complement system may be involved. The aim of this cross-sectional study was to explore whether there is evidence of alternative and/or lectin complement pathway activation in children with IgAVN.

**Methods:**

Children with IgAV were recruited and grouped according to proteinuria: IgAVN or IgAV without nephritis (IgAVwoN). Age and sex-matched healthy controls (HCs) were also recruited. Cross-sectional urine and plasma concentrations of complement factor D (CFD), factor B (CFB), and MBL-associated protease 1 (MASP-1) were performed using commercially available enzyme-linked immunoassays.

**Results:**

A total of 50 children were included (IgAVN, *n* = 15; IgAVwoN, *n* = 20, HCs, *n* = 15). The mean age was 8.5 ± 3.7 years old, male:female ratio was 1:1. Urinary CFD and CFB concentrations were statistically significantly increased in children with IgAVN (3.5 ± 5.4 μg/mmol; 25.9 ± 26.5 μg/mmol, respectively) compared to both IgAVwoN (0.4 ± 0.4 μg/mmol, *P* = 0.002; 9.2 ± 11.5 μg/mmol, *P* = 0.004) and HCs (0.3 ± 0.2 μg/mmol, *P* < 0.001; 5.1 ± 6.0 μg/mmol, *P* < 0.001). No statistically significant difference was reported for the plasma concentrations of CFD and CFB. Urinary MASP-1 concentrations were statistically significantly increased in IgAVN (116.9 ± 116.7 ng/mmol) compared to HCs (41.4 ± 56.1 ng/mmol, *P* = 0.006) and plasma MASP-1 concentrations were increased in IgAVwoN (254.2 ± 23.3 ng/mL) compared to HCs (233.4 ± 6.6 ng/mL, *P* = 0.046).

**Conclusion:**

There is evidence of complement pathway products in the urine of children with IgAVN that warrants further investigation.

## Graphical Abstract

**Graphical Abstract:**
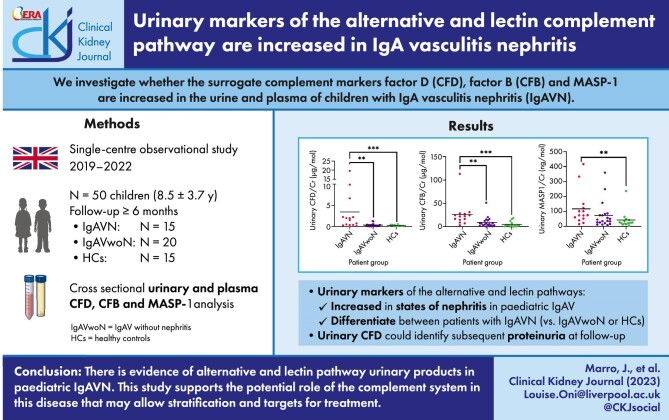

## INTRODUCTION

IgA vasculitis (IgAV, formerly Henoch Schonlein purpura) is the most common type of vasculitis seen in childhood, with an estimated annual incidence of 27.2 per 100 000 children in the United Kingdom [1]. It usually presents as a purpuric non-blanching rash and it is associated with abdominal pain, joint involvement and/or kidney involvement (termed IgAV nephritis, IgAVN) [2]. It is self-limiting in most children but 1–2% will develop chronic kidney disease (CKD) stage 5 [3, 4]. Current clinical practice is to perform urinalysis monitoring for 6 months following presentation to identify any signs of nephritis that typically has an onset of weeks after the initial presentation [5]. This monitoring period offers a window of opportunity for earlier stratification and treatment for those patients who will progress to develop CKD.

The pathophysiology of IgAVN and the mechanisms leading to nephritis are not well understood. It is thought that the mesangial deposition of galactose-deficient forms of IgA1 in the glomerulus triggers an inflammatory response, in a similar way to IgA nephropathy [6]. Emerging evidence supports a potential role of the complement system in this disease [6]. The complement system can be activated through three main routes: the classical pathway, the mannose-binding lectin (MBL) pathway, and the alternative pathway. The classical pathway is activated primarily via IgG/IgM complexes, the lectin pathway by binding to mannose residues on the surface of pathogens, whilst the alternative pathway is constantly activated at low levels and serves as an amplifier. The three pathways converge at C3 activation, which ultimately leads to formation of anaphylatoxins C3a and C5a (a potent neutrophil chemoattractant) and of the membrane-attack complex (MAC), initiating cell-lysis [7].

In IgAV, it is thought that the complement system is activated either through the lectin pathway (LP) or the alternative pathway (AP), with little to no classical pathway activation reported in the literature [6, 8, 9]. Recently, we demonstrated that terminal pathway complement components (C3, C4, C5, C5a) were significantly increased in the urine of children with IgAVN [10]. In addition, we also found increased relative concentrations of CFD in the urine of children with IgAVN in a small exploratory study [11]. We therefore hypothesised that urinary CFD, CFB, and MASP-1 were increased in the urine of children with established IgAVN.

Complement factor D (CFD) and factor B (CFB) not only participate in the initiation of the alternative pathway, but also play a key role in regulating complement activity through the amplification loop, which contributes to about 80% of complement activation regardless of the route used [12, 13]. MBL-associated protease 1 (MASP-1) is a key protease necessary for activation of the LP. There is growing interest in targeting early complement activation and/or complement regulation through these three early components of the complement system, with promising early phase clinical trials in IgA nephropathy [14]. They could also be promising markers of disease activity allowing early identification of high-risks patients.

The aim of this cross-sectional study was to explore whether there is evidence of alternative and/or lectin complement pathway products in children with established IgAVN.

## MATERIALS AND METHODS

### Patient selection

Paediatric participants were recruited at Alder Hey Children's NHS Foundation Trust (Liverpool, UK), between 28 August 2019 and 6 May 2022 as part of a single-centre observational study. Children aged <18 years old at first presentation and diagnosed with IgAV according to the EULAR/PRINTO/PReS 2008 Ankara-endorsed criteria [15] were eligible to take part. Exclusion criteria for this study were as follows: (i) diagnosis of IgAV uncertain or in doubt; (ii) other concurrent inflammatory or kidney condition; (iii) undergoing dialysis; (iv) no samples available in the biobank. Patients were grouped according to the presence of nephritis, into either IgAVN (IgAV with active established nephritis group) or IgAVwoN. Nephritis was defined using the ‘severely increased albuminuria’ definition in KDIGO GN guidelines based on a urinary albumin to creatinine ratio (UACR) of >30 mg/mmol at the time of sampling [16]. The IgAVwoN (group without active nephritis) patient samples were obtained within 4 weeks of IgAV diagnosis whilst no minimum timeline was required for the IgAVN group, providing they had a UACR >30 mg/mmol that was associated with a diagnosis of IgAV. A cohort of age and sex-matched healthy controls (HCs) were recruited for comparison. These children (aged <18 years old) were not taking any regular medications and had no significant past medical history. They were recruited whilst attending for day case surgery or non-inflammatory medical investigations.

### Data collection and definitions

Demographic and clinical data were collected including sex, age at diagnosis, ethnicity, blood pressure (BP), height, serum creatinine, serum albumin (if available), UACR, kidney histology and medication history at the time of sampling to provide the baseline demographic and clinical characteristics. In the patients where a kidney biopsy was conducted, the International Study of Kidney Disease in Children (ISKDC) classification for IgAVN was used to grade the histology [17]. A systolic BP >95th centile for the child's age, sex and height for patients aged less than 16 years old or a measurement >140 mmHg for children aged over 16 years was defined as hypertension [18]. Estimated glomerular filtration rate (eGFR) was calculated as per the Schwartz revised formula [19]. The height value corresponding to the 50th centile for the sex and age was assumed where height data was not available within 2 months of the BP reading or serum creatinine measurement. Normal blood tests results were identified using the age-specific laboratory reference ranges applied at Alder Hey Children's NHS Foundation Trust (Liverpool, UK) [20]. Kidney outcome, UACR and medications were recorded at the time of the last clinical review as of February 2023. For the IgAVN group, kidney outcome was defined as follows: persisting proteinuria as a UACR >30 mg/mmol at last review and resolved proteinuria when UACR <30 mg/mmol. Patients in the IgAVwoN group who did not attend all their routine follow-up appointments were considered to have completed the 6-month follow-up, assuming they would have returned to our tertiary centre with any worsening kidney function. A UACR of 0 mg/mmol was assumed for the patients with a urine dipstick that was negative for protein.

### Sample processing

Urine dipstick testing was used to screen the healthy control samples for bacterial contamination, and they were discarded if they demonstrated positivity for leukocytes, nitrites, blood or > +1 for protein. Urine and plasma samples were centrifuged twice at 300 × *g* for 10 mins, aliquoted, and stored at –80°C. They were defrosted to room temperature on the day of the experiment and vortexed for 10 seconds immediately before use.

### Enzyme-linked immunoassays

As surrogate markers of the alternative and lectin complement pathways, the determination of the urinary and plasma levels of CFD (Biotechne Ltd, Abingdon, UK), CFB (Biotechne Ltd, Abingdon, UK) and MASP-1 (MyBiosource, Inc., San Diego, CA, USA) were performed using commercially available enzyme-linked immunoassays (ELISA) kits as per the manufacturer's instructions. All plates were read immediately after the stop solution was added on a microplate reader at 450 nm with 570 mm wavelength correction on the POLARstar Omega device (BMG LABTECH GmbH, Ortenberg, Germany). For the CFD assay, only the IgAVN urinary samples were diluted 10-fold. This was based on the literature with expected high urinary concentrations of CFD in the IgAVN group. All plasma samples were diluted 400-fold. For the CFB assay, all urine samples were run neat and plasma was diluted 4000-fold. Finally, the MASP-1 assay was run with 4-fold diluted urine samples and 4000-fold diluted plasma samples. Samples that were non-detectable on first measurement were repeated with further adjusted dilution factors accordingly.

### Urinary creatinine quantification

To correct the urinary complement concentrations according to the urine strength, automated quantification of urinary creatinine was performed using a previously described enzymatic method [21] on the Abbott Architect Ci8200 (Abbott, IL, USA) by the Biochemistry Department (Alder Hey Children's NHS Foundation Trust, Liverpool, UK).

### Ethical and regulatory approval

All procedures were conducted in accordance with NIHR Good Clinical Practice, HTA Codes of Practice and the Declaration of Helsinki. The IgA Vasculitis study had HRA and Health and Care Research Wales (HCRW) approval (REC 17/NE/0390). Written informed consent was obtained from parents/guardians and assent obtained from children prior to any study-related procedure.

### Data and statistical analysis

The ELISA data was analysed using MARS Data Analysis Software version 3.32 for Windows (BMG LABTECH GmbH, Ortenberg, Germany). The mean value and standard deviation (SD) of the duplicates were calculated and corrected for dilution and urinary creatinine concentrations. To allow inclusion of all patient samples, any values that remained undetectable due to extreme high or low values, despite further optimisation of dilution factors, were presumed to be either the lowest or highest detectable concentrations of the assay. For each comparison, the fold-change of the mean concentrations were calculated. Data are presented as mean ± SD unless stated otherwise. GraphPad Prism version 8.0 for Windows (GraphPad Software, San Diego, CA, USA) and the Statistical Package for the Social Science (SPSS) version 27.0 software for Windows (IBM Corp, Armonk, NY, USA) were used for the statistical analysis. Shapiro–Wilk test was applied to assess for normality. According to the data distribution the student *t*-test, one-way ANOVA with Tukey's post-hoc test, Mann–Whitney *U* test or Kruskal–Wallis with Dunn–Bonferroni post-hoc test were used to assess for the significance. Pearson's chi square test was applied to continuous variables. Linear correlation was assessed with the Pearson's correlation coefficient following previously published thresholds (<0.30 negligible, 0.30–0.50 low correlation, 0.50–0.70 moderate correlation, 0.70–0.90 strong correlation and 0.90–1.00 very strong correlation) [22]. Receiver operating characteristic (ROC) curves were generated to evaluate the ability of these urinary proteins to discriminate between patients with and without nephritis. Despite the main aim of this study being cross-sectional, an exploratory ROC analysis was conducted on the follow-up data to identify any potential indicators of persisting proteinuria. An area under the curve (AUC) value of <0.7 was deemed non-discriminant, 0.7–0.8 acceptable, 0.8–0.9 excellent and >0.9 outstanding [23]. The significance of the difference between AUCs was assessed using paired-sample AUC difference analysis and optimum cut-offs were identified through classifier evaluation metrics analysis. A *P*-value of <0.05 was considered statistically significant.

## RESULTS

### Demographic and clinical characteristics

A cohort of 50 children (IgAVN, *n* = 15; IgAVwoN, *n* = 20; HCs, *n* = 15) were included in this study. The demographic and clinical characteristics at baseline are shown in Table 1. The mean age of the cohort was 8.5 ± 3.7 years old (range 2.6–17.4), with a male:female ratio of 1:1 and the majority of participants (84%) identified as White British. Children with IgAVwoN were significantly younger (6.4 ± 2.9 years old) than patients in both the IgAVN (9.2 ± 3.6 years old; *P* = 0.047) and HCs groups (10.7 ± 3.2 years old; *P* = 0.001). At baseline, six IgAVN patients had hypertension and seven patients had mildly reduced serum albumin levels (range of abnormal values 22–39 g/L) without evidence of peripheral oedema or clinical significance. Mean eGFR in the IgAVN group was 98.9 ± 18.7 mL/min/1.73 m^2^ compared to 116.8 ± 25.1 mL/min/1.73 m^2^ in IgAVwoN (*P* = 0.065); five children from the IgAVN group had a reduced eGFR between 60 and 90 mL/min/1.73 m^2^, none had a reduced eGFR less than 60 mL/min/1.73 m^2^.

**Table 1:** Patients: characteristics at baseline. ^a^n (%); ^b^mean ± SD. ^1^BP readings were documented in nine patients of the IgAVwoN group. ^2^Serum creatinine and eGFR measurements were available for 19 patients (IgAVN, *n* = 14; IgAVwoN, *n* = 5). UACR: urinary albumin to creatinine ratio; ISKDC: International Study for Kidney Disease in Children Classification; DMARDs: disease modifying anti-rheumatic drugs; DMARDs used in this cohort: hydroxychloroquine, mycophenolate mofetil, azathioprine; eGFR: estimated glomerular filtration rate. Significant *P*-values are highlighted in bold. ^+^*P* < 0.0.5 compared to IgAVwoN; ^***^P < 0.001 compared to HCs; ^+++^*P* < 0.001 compared to IgAVwoN. Due to rounding, percentages may not add up to 100.

	Overall	IgAVN	IgAVwoN	HCs	*P*-value
*n* (%)	50	15	20	15	
Male^a^	25 (50%)	8 (53%)	10 (50%)	7 (47%)	0.936
Age, years^b^	8.5 ± 3.7	9.2 ± 3.6^+^	6.4 ± 2.9^***^	10.7 ± 3.2	**<0.001**
*Ethnicity*					
White british	42 (84%)	13 (87%)	18 (90%)	11 (73%)	
African	3 (6%)	0 (0%)	0 (0%)	3 (20%)	
Chinese	2 (4%)	0 (0%)	2 (10%)	0 (0%)	
Any other Asian background	1 (2%)	0 (0%)	0 (0%)	1 (7%)	
Not stated	2 (4%)	2 (13%)	0 (0%)	0 (0%)	
**Sampling**					
Sample type					
*Urine and blood^a^*	30 (60%)	10 (67%)	5 (25%)	15 (100%)	
*Urine only*^a^	19 (38%)	4 (27%)	15 (75%)		
*Blood only*^a^	1 (2%)	1 (7%)	0 (0%)		
Weeks from diagnosis to sampling^b^	17.2 ± 51.9	37.9 ± 75.7	1.7 ± 0.9		**<0.001**
**Kidney involvement**					
Hypertension^a, 1^	7 (29%)	6 (40%)	1 (11%)		0.132
Serum creatinine μmol/L^b,2^	51.9 ± 16.4	53.1 ± 14.7	48.6 ± 22.1		0.643
Serum albumin g/L^b,3^	37.5 ± 5.6	35.8 ± 5.1	43.0 ± 3.7		**0.012**
eGFR mL/min/1.73m^2 b,2^	104.6 ± 21.9	98.9 ± 18.7	116.8 ± 25.1		0.065
UACR mmol/mg^b^	172.9 ± 422.6	401.1 ± 579.9	1.9 ± 5.1		
Urinary creatinine mmol/L^b^	8.4 ± 7.1	6.2 ± 4.8	7.4 ± 7.2	11.6 ± 8.1	0.066
**Biopsy proven nephritis^a^**					
*ISKDC Grade*					
II^a^		1 (11%)			
IIIa^a^		1 (11%)			
IIIb^a^		6 (67%)			
IV^a^		1 (11%)			
**Medications**					
Corticosteroids^a^	6 (12%)	6 (40%)	0 (0%)		
DMARDs^a^	5 (10%)	5 (33%)	0 (0%)		
ACE inhibitor^a^	1 (2%)	1 (7%)	0 (0%)		
**Follow-up**					
*n*, %	33 (100%)	13 (39%)	20 (61%)		
Months^b^	17.2 ± 17.4	29.6 ± 21.4	9.2 ± 7.0		**<0.001**
UACR at last review^b^	15.6 ± 43.1	39.6 ± 62.6	0.0 ± 0.0		**<0.001**
Outcome^a^		Resolved proteinuria, 6 (46%)			
		Persisting proteinuria, 7 (54%)			

Of the IgAVN group, nine (60%) had a kidney biopsy with nephritis demonstrated histologically, all of them demonstrated IgA predominant immune deposits and eight (89%) patients had C3 positivity on immunofluorescence. IgG deposits were present in three (33%) patients and fibrinogen in one. No patients were found positive for IgM or C4. There was a significant difference in the time from diagnosis from sampling between the two groups, with IgAVN samples being obtained at a later time point as expected in the evolution of nephritis.

The mean follow-up captured in the cohort was 17.2 ± 17.4 months (range 6.0–79.0 months). Two IgAV patients were excluded from the follow-up analysis (care being handed over to another centre, *n* = 1; no urinary sample but only plasma sample available at baseline, *n* = 1). None of the IgAVwoN patients evolved into IgAVN during follow-up (range of follow-up for IgAVwoN group was 6.0–32.0 months). In terms of kidney outcomes within the IgAVN group, proteinuria resolved in six (46%) patients whilst seven (54%) had persisting proteinuria. At last clinical review, three (21%) patients remained on corticosteroid treatment, three (21%) were being treated with mycophenolate mofetil (MMF) and three (21%) were prescribed the angiotensin converting enzyme inhibitor (ACEi) lisinopril.

### Cfd

**Figure 1:**
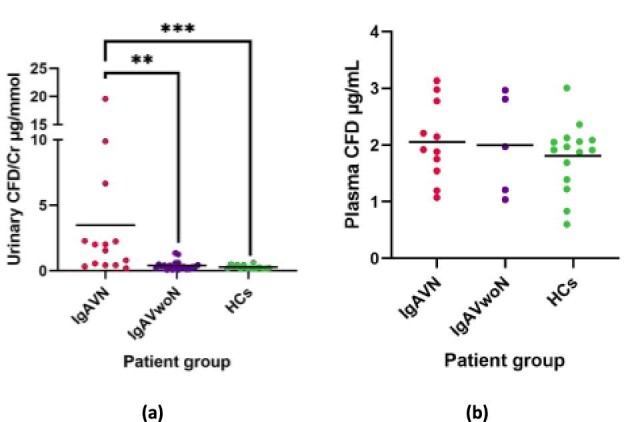
CFD immunoassay results with mean lines. (**a**) Urinary CFD concentrations between patients with IgAVN, IgAVwoN and HCs corrected for urinary creatinine (Cr); (**b**) Plasma CFD concentrations between patients with IgAVN, IgAVwoN and HCs. *P < 0.05, **P < 0.01, ***P < 0.001.

Urinary CFD concentrations were significantly increased in patients with IgAVN (3.5 ± 5.4 μg/mmol) compared to both IgAVwoN (0.4 ± 0.4 μg/mmol; 9.0-fold increase; *P* = 0.002) and HCs (1.8 ± 0.6 μg/mL; 12.9-fold; *P* < 0.001) (Fig. 1a). No significant difference was observed between IgAVwoN and HCs. Three outliers were noted in the IgAVN group. As shown in Fig. 1b, no statistically significant difference or trend was identified for the plasma concentrations of CFD (IgAVN 2.1 ± 0.7 μg/mL; IgAVwoN 2.0 ± 0.9 μg/mL; *P* = 0.490).

### Cfb

**Figure 2:**
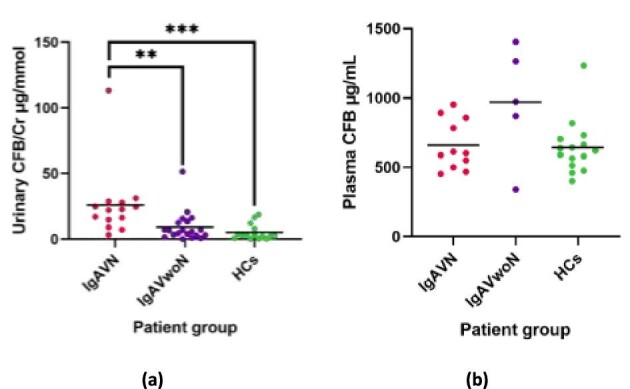
CFB immunoassay results with mean lines. (**a**) Urinary CFD concentrations between patients with IgAVN, IgAVwoN, and HCs corrected for urinary creatinine (Cr); (**b**) Plasma CFB concentrations between patients with IgAVN, IgAVwoN, and HCs. *P < 0.05, **P < 0.01, ***P < 0.001.

Urinary CFB concentrations were also significantly greater in IgAVN (25.9 ± 26.5 μg/mmol) than in IgAVwoN (9.2 ± 11.5 μg/mmol; 2.8-fold; *P* = 0.004) and HCs (5.1 ± 6.0 μg/mmol; 5.0-fold; *P* < 0.001). Urinary CFB concentrations did not significantly differ between IvAVwoN and HCs (*P* = 0.608). One outlier was noted in IgAVN and one in IgAVwoN, as demonstrated in Fig. 2a. In a similar way to CFD, no significant difference was observed in the CFB plasma concentrations depending on nephritis status (IgAVN 659.8 ± 179.5 μg/mL; IgAVwoN 970.8 ± 413.6 μg/mL; *P* = 0.170) (Fig. 2b).

### Masp-1

**Figure 3:**
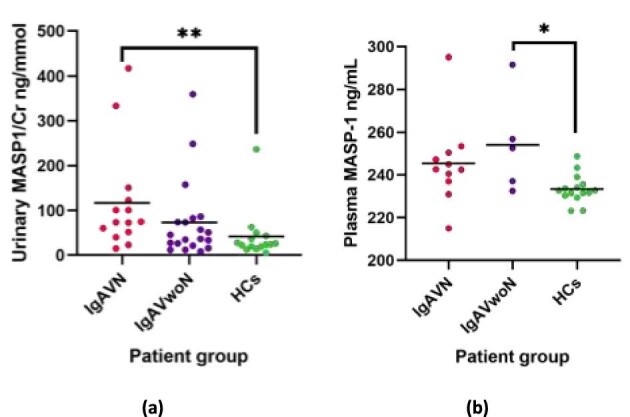
MASP-1 immunoassay results with mean lines. (**a**) Urinary MASP-1 concentrations between patients with IgAVN, IgAVwoN, and HCs corrected for urinary creatinine (Cr); (**b**) Plasma MASP-1 concentrations between patients with IgAVN, IgAVwoN, and HCs. *P < 0.05, **P < 0.01.

Similar findings were reported for MASP-1 with a 2.8-fold increase in urinary concentrations in the patients with IgAVN (116.9 ± 116.7 ng/mmol) when compared to the cohort of HCs (41.4 ± 56.1 ng/mmol; *P* = 0.006); but did not reach statistical significance between IgAVN and IgAVwoN (73.0 ± 88.4 ng/mmol; *P* = 0.240) and between IgAVwoN and HCs (Fig. 3a). Notable outliers were present across all groups. Plasma MASP-1 concentrations were also increased in the IgAVwoN group compared to HCs (254.2 ± 23.3 ng/mL v 233.4 ± 6.6 ng/mL; *P* = 0.046). A non-significant trend was observed with increased plasma MASP-1 concentrations between IgAVN and the HCs group (245.4 ± 19.5 ng/mL *v* 233.4 ± 6.6 ng/mL; *P* = 0.062) (Fig. 3b).

### Correlation with other markers and ability to identify patients with nephritis

Urinary complement components, plasma components and UACR were correlated to each other to identify any association in the patients with IgAV. It was found that urinary CFD and MASP-1 were positively associated with proteinuria, with urinary CFD demonstrating a strong correlation (r = 0.85; *P* < 0.001) and MASP-1 a moderate correlation (r = 0.54; *P* < 0.001). Interestingly, urinary MASP-1 and CFD levels were also moderately positively correlated with each other (r = 0.41; *P* = 0.015). No other significant correlation with the eGFR or any other variables were reported. No significant associations were found between histological findings and complement concentrations (although the number of patients in this group was very low).

**Figure 4:**
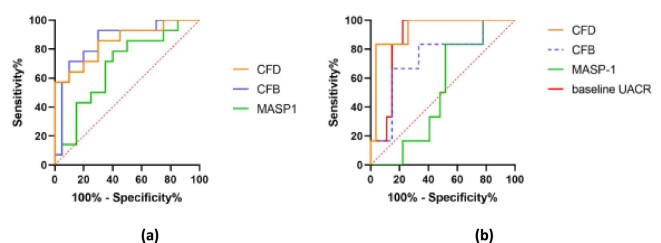
Receiver operating characteristic (ROC) curve analysis for individual urinary complement concentrations assessing their effectiveness at distinguishing between IgAVN and IgAVwoN (**a**); for individual baseline urinary complement concentrations and baseline UACR assessing their effectiveness at identifying subsequent proteinuria during follow-up (**b**).

ROC curves were generated to determine the ability of these complement proteins to differentiate between patients with and without nephritis (UACR of >30 mg/mmol) (Fig. 4a). Urinary CFD (AUC 0.850; 95% CI [0.715–0.985]; *P* < 0.001) and CFB (AUC 0.854; 95% CI [0.719–0.988]; *P* < 0.001) were individually excellent to discriminate patients with IgAVN, whereas MASP-1 did not reach statistical significance (AUC 0.682; 95% CI [0.499–0.865]; *P* = 0.074).

### Exploratory analysis of the potential of urinary complement components to identify subsequent proteinuria

ROC curves were generated to determine whether these urinary complement proteins could be indicators of subsequent proteinuria at follow-up (Fig. 4b). A total of 33 out of the 35 IgAV patients were included in this exploratory analysis (persisting proteinuria, *n* = 6; no proteinuria, *n* = 27). The ‘no proteinuria’ group consisted of seven IgAVN patients who had resolved nephritis over time alongside 20 IgAVwoN patients who never developed proteinuria during the follow-up period. The baseline urinary CFD (AUC 0.932; 95% CI [0.835–1.000]; *P* = 0.001) and baseline UACR (AUC 0.8704; 95% CI [0.7488–0.9919]; *P* = 0.005) were both excellent at identifying those patients with persisting proteinuria (UACR >30 mg/mmol) over time, with urinary CFD demonstrating a higher AUC than baseline UACR. However, the AUC difference was not statistically significant based on paired-sample AUC difference analysis (z = 0.923, *P* = 0.356). The identified optimum cut-offs were 1.78 μg/mmol for baseline urinary CFD (sensitivity: 83.3%, specificity 96.3%) and 98.1 mg/mmol for baseline UACR (sensitivity: 100%, specificity 77.8%). Urinary CFB (AUC 0.741; 95% CI [0.514–0.968]; *P* = 0.069) and MASP-1 (AUC 0.512; 95% CI [0.308–0.717]; *P* = 0.926) did not reach statistical significance.

## DISCUSSION

The aim of this cross-sectional study was to explore whether there is evidence of alternative and/or lectin complement pathway activation in children with established IgAVN, through the measurement of urinary and plasma concentrations of CFD, CFB, and MASP-1. This study used a cohort of children with IgAV and healthy controls to report that complement urinary products that may reflect activation of predominantly the alternative (AP) but also lectin pathways (LP) are found in the urine of patients with IgAVN. To our knowledge, this is the first study assessing these specific complement components in paediatric IgAVN. This follows on from our previous work that identified increased urinary concentrations of terminal pathway products [10] and confirms our previous exploratory findings that included a relative increased expression of CFD in IgAVN when using a broad spectrum protein array [11].

In the current study, all three urinary complement products were increased in IgAVN, although MASP-1 was only significantly increased when compared to HCs, regardless of the plasma concentrations. Our findings suggest that these complement products may be able to differentiate patients with nephritis on a cross-sectional basis as illustrated by the excellent AUCs for urinary CFD and CFB. CFD and CFB participate in both alternative pathway initiation and complement regulation, with CFD described as the rate-limiting enzyme of the complement system, hence making selective CFD inhibition very attractive [12]. MASP-1 is needed for LP activation after binding of LP-pattern recognition molecules (PRMs, mainly MBL) to carbohydrate residues on the surface of pathogens [14]; and human IgA has been shown to activate the complement system via the LP *in vitro* [24]. Although LP-associated PRMs can bind IgA and GalNac residues [25], it is unknown whether the exposed glycans are the sole reason of complement system activation in IgA-mediated glomerulonephritis [26].

Currently, complement activation is recognised in patients with IgAVN mainly through C3 histological deposition on immunofluorescence [2, 27], although its pathogenic relevance has remained unclear. In IgAV, growing evidence supports the complement system is activated via the AP and LP [6, 8] mainly relying on histological evidence of complement deposition and increased circulating levels [28–30]. Histological deposition of complement components of the AP, LP and terminal pathways have been previously reported in the kidney histology in IgAV [28, 30–32], but without necessarily correlating with outcomes [33–35]. It has also been proposed that genetic polymorphisms of complement factors, especially of the AP, may play a role in determining the severity of the kidney phenotype in IgA-mediated nephritis [26, 36, 37].

In IgA nephropathy, increased urinary complement factors are reported [38] and may be of prognostic use, as urinary C4d and MBL levels positively correlated with the percentage of crescents [39] and similar findings have been reported for urinary CFH [40]. In another study, urinary CFH was associated with kidney function decline and was superior in predicting outcomes compared to both eGFR and proteinuria for adults with IgA nephropathy [41]. In other glomerulonephritides, such as lupus nephritis, ANCA-associated vasculitis nephritis or focal segmental glomerulosclerosis, urinary evidence of alternative pathway activation has also been reported in the literature, indicating that complement activation may not be unique to IgA-mediated kidney diseases [42–44]. Wen and colleagues also previously reported significantly increased urinary MBL concentrations in patients with IgA nephropathy compared to a group with non-proliferative CKD, suggesting the LP may be more specific of IgA-related pathogenesis [38]. When compared to HCs, patients with minimal change disease and nephrotic range proteinuria did not have elevated urinary complement levels [45]. As such, urinary complement profiles may vary depending on disease aetiology.

One concern about any urine biomarker is how they arise in the urine and their relationship with proteinuria, especially as we report a correlation between CFD and MASP-1 and the degree of proteinuria. Complement products are usually produced by the liver (or adipocytes for CFD) [12]; however, it has been demonstrated that kidney cells, specifically kidney tubular epithelial cells, can synthesise all complement proteins [46–48]. In addition, CFD is usually reabsorbed by the tubular cells and previous evidence has shown urinary markers of tubular inflammation are raised in children with IgAVN [11, 49]. It is important to note that these complement products are not specific to kidney inflammation and could also reflect broader extra-renal mechanisms. Thus, it is not possible to know from this preliminary data whether the increased urinary concentrations reported in this study are due to an inflamed leaking glomerulus (hence their relationship to proteinuria), to surplus systemic amounts, over-excretion from tubular damage or due to excess intrinsic production. However, our exploratory cohort is the first to report that urinary CFD may be an indicator of subsequent persisting proteinuria during follow-up and this warrants further investigation.

Therapeutic targeting of the complement system constitutes a novel, attractive approach for treating this disease. Currently, several clinical trials are assessing the efficacy of selective complement inhibitors in IgA nephropathy, with promising results showing a reduction in proteinuria and inhibition of route-specific complement activation reported for CFB and MASP-2 inhibitors [50, 51]. To date, there is only one case report of the use of a complement inhibitor (MASP-2 inhibitor narsoplimab) in a young woman with rapidly progressive IgAVN, which resulted in delayed kidney function decline and sustained reduction in lectin pathway activation [52].

There are several limitations to this study that include its small size and cross-sectional analysis. Additionally, the samples were collected pragmatically with blood samples only collected at the time of clinical sampling. This meant that there were fewer plasma samples Our definition of nephritis also solely relied on the degree of proteinuria. Another limitation to consider regards the significant difference in sampling time between the children with established IgAVN and IgAVwoN, and future studies will look at capturing the evolution of nephritis using early urine samples. The cross-sectional focus of this study did not allow for assessment of the time of onset in relation to the disease evolution to illustrate the true prognostic value of these urinary proteins; this will be the focus of future longitudinal studies. The study would have benefitted from a disease-control group to assess any specificity to IgAVN. Despite these limitations, our findings, alongside existing evidence, supports further studies to explore the complement pathway as a way to stratify and therapeutically target IgAVN.

## CONCLUSIONS

This study supports the potential role of the complement system in the pathophysiology of IgAVN that may act as a therapeutic target. Further large longitudinal studies are required to fully evaluate their timing of onset, relationship to proteinuria and role in the evolution of nephritis for children with IgAV.

## Data Availability

Data will be shared on reasonable request to the corresponding author.
